# Cuproptosis and anti-tumor immunity: bidirectional regulation, controversies, and translational prospects

**DOI:** 10.3389/fimmu.2026.1932651

**Published:** 2026-09-22

**Authors:** Xiangxiang Ren, Tianhao Xie, Xiaoshi Jin, Meng Zhang

**Affiliations:** 1 Department of General Surgery, Affiliated Hospital of Hebei University, Baoding, Hebei, China; 2 Department of Dermatology, Affiliated Hospital of Hebei University, Baoding, Hebei, China

**Keywords:** anti-tumor immunity, copper ionophores, copper metabolism, cuproptosis, ferroptosis, immune checkpoint inhibitors, immunogenic cell death, tumor microenvironment

## Abstract

Cuproptosis is a copper-dependent form of regulated cell death driven by copper binding to lipoylated tricarboxylic acid (TCA) cycle proteins. This interaction triggers aberrant protein aggregation, Fe-S cluster protein degradation, and mitochondrial proteotoxic stress, and its unique metabolic dependency distinguishes cuproptosis from apoptosis and ferroptosis. This review examines the bidirectional regulation between cuproptosis and anti-tumor immunity. Cuproptosis can promote dendritic cell maturation and CD8+ T cell-mediated immunity through immunogenic cell death (ICD), damage-associated molecular pattern release, and cGAS-STING pathway activation. Activated CD8+ T cells may in turn enhance tumor cell cuproptosis sensitivity via IFN-γ-STAT1-IRF1-driven FDX1 upregulation, forming a positive feedback loop. Conversely, direct copper toxicity toward T cells and NK cells, spatial heterogeneity of intratumoral copper distribution, and potential immunosuppressive cytokine release may counterbalance these immunostimulatory effects. We outline core controversies regarding the universality of cuproptosis immunogenicity, the immune activation window of copper ions, and the clinical utility of cuproptosis biomarkers. Translational strategies including copper ionophore-immunotherapy combinations, copper-based nano-delivery systems, and copper metabolism-based patient stratification are discussed. The cuproptosis-immunity intersection remains at an early stage; key mechanistic findings require independent validation before clinical translation.

## 1 Introduction

Copper is an essential trace element involved in electron transport, redox homeostasis, angiogenesis, and neural development (1). Experimental evidence has established its role in tumor biology: tumor cells display an abnormally high demand for copper, copper accumulation correlates with tumor proliferation, metastasis, and angiogenesis, and copper functions as an obligate cofactor for certain oncogenic signaling pathways, including BRAF-MEK-ERK (2, 3). However, copper exerts bidirectional effects on cell fate -- both copper deficiency and copper overload can cause cellular dysfunction and death, and tumor cells must tightly regulate intracellular copper homeostasis to survive.

Prior to Tsvetkov et al. (4), copper-induced cell death was attributed primarily to copper-mediated oxidative stress and reactive oxygen species (ROS) accumulation, a nonspecific toxic effect. Using genome-wide CRISPR-Cas9 screening, Tsvetkov et al. identified a previously unknown mechanism: copper ions directly bind lipoylated mitochondrial proteins (particularly DLAT), inducing their aberrant aggregation while destabilizing and degrading Fe-S cluster proteins, ultimately triggering proteotoxic stress (4). They termed this copper-specific, apoptosis-, ferroptosis-, and pyroptosis-distinct mode of cell death “cuproptosis,” thereby adding a new metal-dependent regulated cell death category to the Nomenclature Committee on Cell Death (NCCD) classification system (4, 5). This discovery established a mechanistic basis for copper metabolism-targeted cancer therapy.

If cuproptosis is a specifically inducible programmed death pathway, then employing copper ionophores (e.g., elesclomol, disulfiram) to selectively trigger cuproptosis in tumor cells becomes a therapeutic strategy. However, the value of cuproptosis in cancer therapy depends on both its direct tumor cell killing and its impact on host anti-tumor immune responses: the mode by which tumor cells die determines whether the immune system perceives and responds to danger signals or remains tolerant to the death event (6–8).

The central question of this review is: What role does cuproptosis play in anti-tumor immunity? Is it an ally that activates adaptive immunity through immunogenic cell death, or an impediment to immune responses due to the bidirectional effects of copper ions on immune cells? Several excellent reviews have covered cuproptosis mechanisms (6, 7, 9–13) and its general role in cancer therapy. Comprehensive overviews have covered cuproplasia and cuproptosis in cancer (7), molecular mechanisms and therapeutic applications (8), and copper homeostasis in cancer immunity (9). This review differs by focusing on the bidirectional regulation between cuproptosis and anti-tumor immunity, providing an evidence-level-annotated assessment of current experimental data, and identifying concrete experimental gaps that must be closed before clinical translation. We examine the current understanding of bidirectional regulation, clarify key controversies, and discuss translational pathways and bottlenecks. The cuproptosis-immunity intersection remains in its early stages, and certain mechanistic findings, particularly the positive feedback loop described in Section 3, derive from a single study (10) and await systematic validation by multiple independent laboratories. Evidence levels and uncertainties are explicitly noted throughout relevant sections (**Figure 1**).

**Figure 1:**
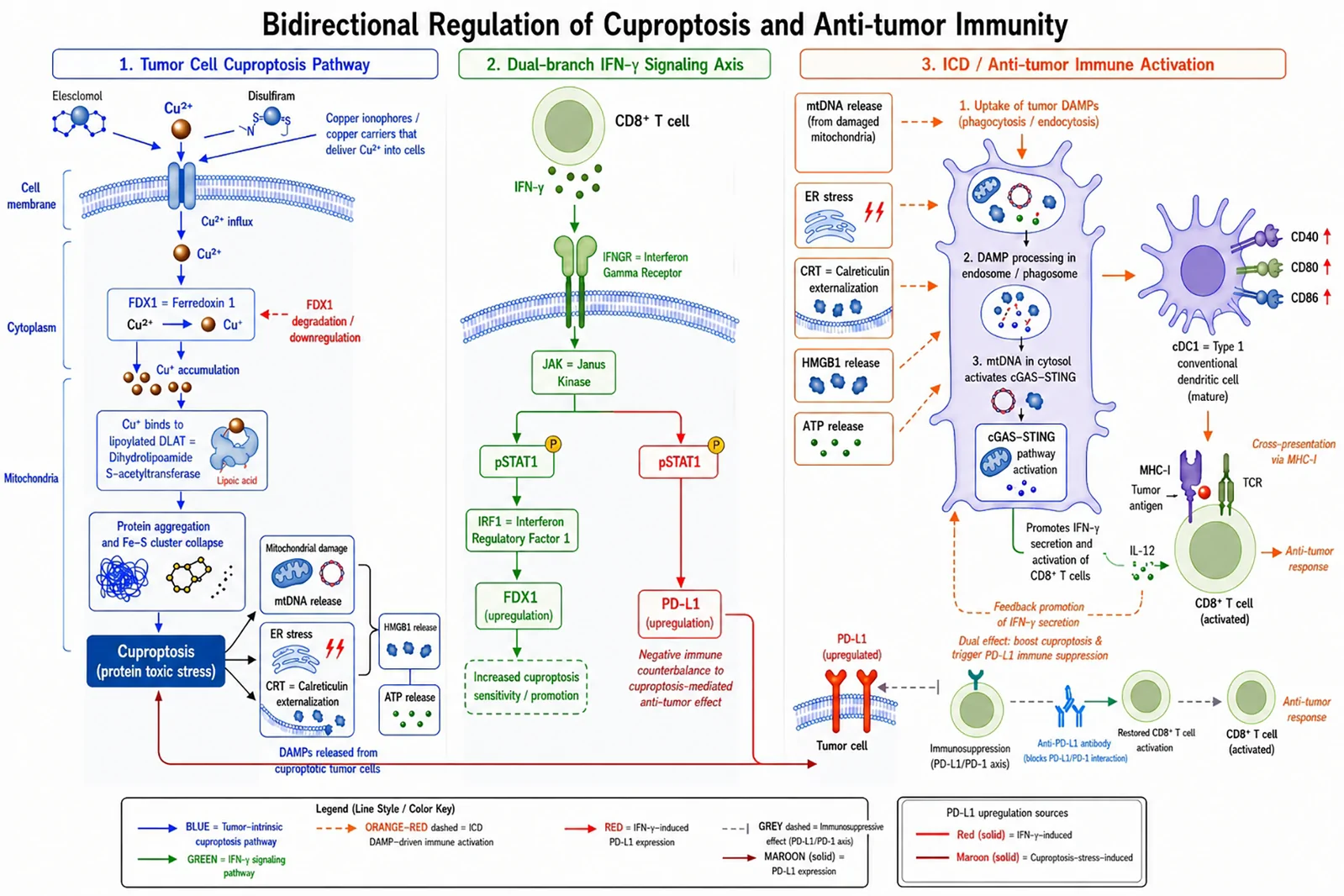
Bidirectional regulation of cuproptosis and anti-tumor immunity. Copper ionophores import Cu2+; FDX1 reduces Cu2+ to Cu+, which binds lipoylated DLAT, inducing protein aggregation, Fe-S cluster collapse, and cuproptosis. mtDNA release activates cGAS-STING, and ER stress triggers CRT exposure and HMGB1/ATP release, promoting DC maturation and CD8+ T cell activation. IFN-γ from CD8+ T cells drives FDX1 transcription via JAK-STAT1-IRF1 (positive feedback). Dashed lines: copper toxicity toward immune cells; PD-L1 upregulation-based combinatorial strategies.

## 2 Molecular mechanisms of cuproptosis

### 2.1 Intracellular copper homeostasis

Strict regulation of intracellular copper levels is required for normal cellular function. Copper enters cells via the plasma membrane copper transporter SLC31A1 (also known as CTR1), while copper efflux depends on two P-type ATPases: ATP7A and ATP7B (1). In the cytoplasm, copper ions do not exist in free form; rather, a suite of copper chaperones delivers them to specific target proteins: CCS (copper chaperone for SOD1) delivers copper to superoxide dismutase 1 (SOD1), COX17 transports copper to mitochondrial respiratory chain complex IV (cytochrome c oxidase), and ATOX1 transfers copper to ATP7A/ATP7B for efflux or delivery to the secretory pathway (2, 11). Reviews have described the molecular framework of the copper homeostasis regulatory network (9, 12).

Tumor cells frequently exhibit copper homeostasis dysregulation. Studies show upregulation of SLC31A1 in multiple solid tumors, including breast, lung, and colorectal cancers, with intracellular copper levels exceeding those of normal tissues (2, 13). This copper accumulation results partly from tumor metabolic reprogramming, yet simultaneously constitutes a therapeutic vulnerability: tumor cell copper buffering systems operate near capacity, and further exogenous copper influx may exceed buffering thresholds, triggering cuproptosis (3).

### 2.2 Upstream signals of cuproptosis: the FDX1-LIAS-lipoylation axis

Cuproptosis initiation critically depends on the mitochondrial lipoylation metabolic pathway (4). Lipoylation is a highly conserved post-translational modification occurring primarily on subunits of two key TCA cycle enzyme complexes: dihydrolipoamide S-acetyltransferase (DLAT, the E2 subunit of the pyruvate dehydrogenase complex) and dihydrolipoamide S-succinyltransferase (DLST, the E2 subunit of the alpha-ketoglutarate dehydrogenase complex). Lipoylation is catalyzed by lipoic acid synthase (LIAS), whose Fe-S cluster assembly in turn depends on ferredoxin 1 (FDX1) reducing Cu2+ to the more reactive Cu+. Subsequent reviews have confirmed and expanded the core mechanism of cuproptosis (9–13).

Thus, FDX1-LIAS-lipoylation constitutes a functional axis whose activity level determines cellular sensitivity to cuproptosis. When copper ionophores (e.g., elesclomol) import large quantities of copper, FDX1 first reduces Cu2+ to Cu+, which then directly binds lipoylated DLAT and DLST, causing conformational changes and hydrophobic exposure, ultimately leading to aberrant protein aggregation and precipitation (4, 14). FDX1 itself is a direct effector target of cuproptosis: FDX1 expression levels positively correlate with cellular sensitivity to cuproptosis, and FDX1 knockout confers complete resistance to cuproptosis (4, 15).

### 2.3 Execution phase of cuproptosis: protein aggregation and Fe-S cluster collapse

Cuproptosis execution proceeds through two parallel cascades: proteotoxic stress and Fe-S cluster protein loss. Copper-induced aggregation of lipoylated DLAT is the hallmark molecular event of cuproptosis (16). This aggregation causes loss of pyruvate dehydrogenase complex function, blocking the TCA cycle and oxidative phosphorylation, while the aggregated protein masses themselves trigger the mitochondrial unfolded protein response (UPRmt) and proteotoxic stress (4, 17). Transmission electron microscopy reveals mitochondrial morphological abnormalities in cuproptotic cells, including cristae loss and matrix densification; these ultrastructural features were first described in Tsvetkov et al.’s original study and subsequently confirmed in reviews (4, 18).

The other critical event is a decline in mitochondrial Fe-S cluster protein levels. FDX1 not only participates in Cu2+ to Cu+ reduction but is itself an Fe-S cluster protein (19). Excess copper ions disrupt Fe-S cluster biosynthesis and/or stability, leading to degradation of multiple Fe-S cluster proteins, including LIAS, aconitase (ACO2), and NADH dehydrogenase subunits (4). The consequences are manifold: LIAS loss creates positive feedback that further impairs lipoylation capacity; widespread respiratory chain dysfunction exacerbates the mitochondrial metabolic crisis, synergizing with proteotoxic stress to drive cells toward irreversible death (**Table 1**).

**Table 1:** Key regulatory molecules of cuproptosis and their roles in antitumor immunity.

Molecule	Category	Role in cuproptosis	Relevance to antitumor immunity
FDX1	Reductase/Fe-S protein	Reduces Cu2+ to Cu+; rate-limiting for cuproptosis (4)	Upregulated by IFN-γ-STAT1-IRF1; links T cell activity to cuproptosis sensitivity (10)
LIAS	Lipoyltransferase	Catalyzes lipoylation of DLAT/DLST (4)	Lipoylation status may predict tumor ICD responsiveness
DLAT	PDH E2 subunit	Cu+ binding target; aggregation triggers proteotoxic stress (4, 16)	Aggregated DLAT may serve as neoantigen or DAMP (10)
SLC31A1	Copper importer	Major copper uptake transporter (1, 50)	Overexpressed in tumors; gate for ionophore-delivered copper (13)
ATP7A/ATP7B	Copper exporters	Copper efflux pumps (9, 11)	Downregulation enhances cuproptosis sensitivity
GPX4	Selenoprotein peroxidase	Detoxifies lipid peroxides (21, 23)	GSH depletion promotes cuproptosis but also ferroptosis
MT1/MT2	Metallothioneins	Copper buffering and sequestration (20)	High expression may suppress cuproptosis; p53 target
GSH	Tripeptide antioxidant	Copper chelation; GPX4 cofactor (22)	Depletion links cuproptosis and ferroptosis (21)
cGAS-STING	DNA sensing pathway	Activated by mtDNA release (36–38)	Core ICD-to-adaptive-immunity bridge (10)
IFN-γ	Type II interferon	Transcriptional activator of FDX1 (45, 46)	Positive feedback loop driver (10)
STAT1	Transcription factor	Mediates IFN-γ signaling (46)	FDX1 transcriptional regulation
IRF1	Transcription factor	Downstream of STAT1 (46)	Directly binds FDX1 promoter (10)
PD-L1	Immune checkpoint	Upregulated upon cuproptosis (10, 13)	ICI combination target

### 2.4 Regulatory networks of cuproptosis and cross-talk with ferroptosis

Cuproptosis does not operate in isolation but is embedded within cell death regulatory networks (5, 20). Several regulatory nodes have been identified: (1) GLUT1/SLC31A1 axis: Glucose transporter 1 (GLUT1) expression correlates with copper uptake, and tumor cells with high glycolytic activity may exhibit greater cuproptosis sensitivity. (2) GPX4 and the glutathione system: Glutathione (GSH) and metallothioneins (MTs) together constitute an important intracellular copper buffering system; GSH depletion increases free copper levels and promotes cuproptosis. However, GSH is also a core negative regulator of ferroptosis (as a cofactor for GPX4), meaning that intervention in the GSH/GPX4 system may simultaneously affect both cuproptosis and ferroptosis (23–26). (3) Copper chaperone network: Expression levels of copper chaperones such as ATOX1 and CCS influence the compartmentalized distribution and bioavailability of intracellular copper, thereby modulating the cuproptosis threshold (9, 12). (4) Ubiquitin-proteasome system: Copper-induced protein aggregates can activate ubiquitination degradation pathways, and the functional status of this system may affect the capacity to clear toxic protein aggregates (16). p53 regulation of cuproptosis is under investigation; preliminary evidence suggests p53 may indirectly influence cuproptosis sensitivity through transcriptional regulation of copper-binding proteins (e.g., metallothioneins), but the specific mechanism and directionality require elucidation.

Beyond the GSH/GPX4 node, additional intersections between cuproptosis and ferroptosis (21, 22) are worth noting. Iron-sulfur cluster proteins, whose degradation is a hallmark of cuproptosis (4),also participate in iron sensing and ferroptosis regulation (23, 24). Mitochondrial dysfunction, a shared feature of both death modes, creates the potential for convergent vulnerability: cuproptosis-induced respiratory chain collapse could deplete NADPH and reduce GPX4 activity, while ferroptotic lipid peroxidation may damage mitochondrial membranes containing lipoylated proteins. The question of whether simultaneous induction of cuproptosis and ferroptosis produces additive, synergistic, or antagonistic immune effects in the tumor microenvironment remains unexplored. (**Figure 2**) Ferroptosis inducers have been shown to enhance ferroptosis inducers enhanced cuproptosis in liver cancer models (25), but the immunological consequences of dual induction have not been systematically examined (**Table 2**).

**Figure 2:**
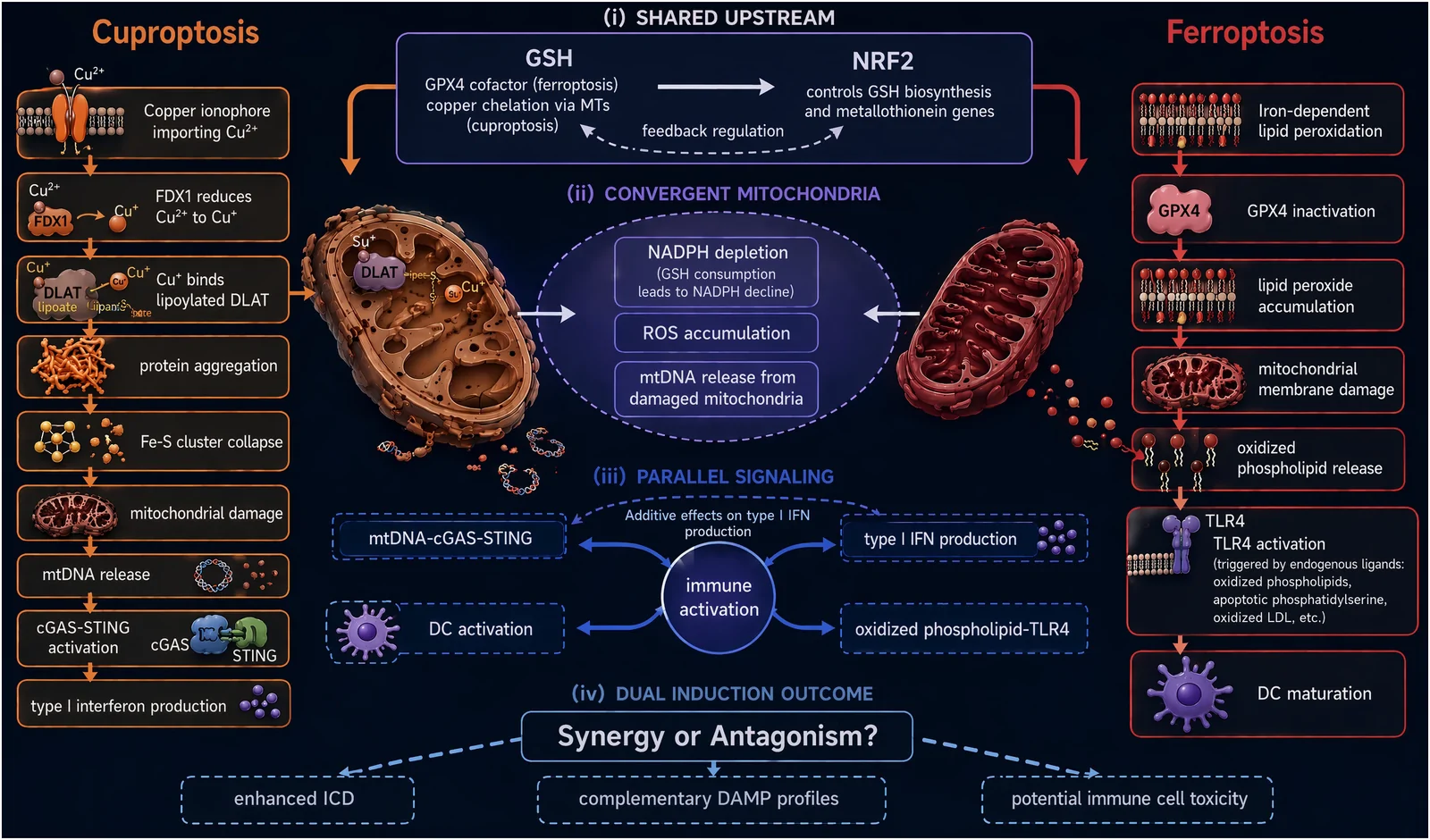
Schematic illustration of the crosstalk between cuproptosis and ferroptosis. The four levels are: (i) shared upstream regulators (GSH, NRF2); (ii) convergent mitochondrial damage (DLAT aggregation, Fe-S cluster loss, lipid peroxidation -> NADPH depletion, ROS, mtDNA release); (iii) partially overlapping signaling (mtDNA-cGAS-STING-type I IFN; oxidized phospholipid-TLR4 -> DC activation); (iv) potential synergy/antagonism of dual induction.!!!See Section 2.4 for detailed discussion of each level.

**Table 2:** Systematic comparison of cuproptosis and ferroptosis in tumor immunology.

Feature	Cuproptosis	Ferroptosis
Year defined	2022 (Tsvetkov et al.) (4)	2012 (Dixon et al.) (24)
Primary trigger	Copper overload, lipoylated protein binding	Iron-dependent lipid peroxidation
Core mechanism	Lipoylated protein aggregation + Fe-S cluster protein degradation	Lipid peroxide accumulation, GPX4 inactivation
Key molecules	FDX1, LIAS, DLAT, DLST, SLC31A1	GPX4, SLC7A11, ACSL4, LPCAT3, ALOXs
Mitochondrial morphology	Cristae loss, matrix densification	Shrinkage, increased membrane density
Primary DAMPs	mtDNA, HMGB1, ATP; CRT exposure	HMGB1, ATP; oxidized phospholipids
ICD signaling pathway	mtDNA-cGAS-STING-type I IFN; ER stress-CRT	Lipid peroxide-TLR4; ACSL4-LPCAT3-ALOXs axis
DC activation mode	Type I IFN-driven cDC1 cross-presentation	Partially TLR4-dependent
IFN-γ feedback loop	STAT1-IRF1-FDX1 transcriptional upregulation	SLC7A11/SLC3A2 downregulation, cystine deprivation
ICI synergy mechanism	PD-L1 upregulation; ICD-enhanced T cell priming	PD-L1 downregulation; enhanced T cell infiltration
Immune memory evidence	Limited (MC38 model, single study)	Validated in multiple independent vaccination models
Therapeutic agents	Elesclomol-Cu, disulfiram-Cu	Erastin, RSL3, FIN56, GPX4 inhibitors

MDA, malondialdehyde; 4-HNE, 4-hydroxynonenal; PE, phosphatidylethanolamine; mtDNA, mitochondrial DNA; HMGB1, high mobility group box 1; CRT, calreticulin; ER, endoplasmic reticulum; cDC1, conventional dendritic cell type 1; ICI, immune checkpoint inhibitor.

## 3 Cuproptosis-mediated promotion of anti-tumor immunity

### 3.1 Cuproptosis as immunogenic cell death: tumor cell-intrinsic events

The core immunological characterization of cuproptosis as ICD, including CyTOF-based immune profiling, DC dependence evidence, and the prophylactic vaccination protection model, derives from a single comprehensive study by Lei et al. (10). The ICD component has received preliminary independent corroboration in hepatocellular carcinoma (33) and osteosarcoma (34) models, but the complete immunological landscape described below remains to be validated across independent laboratories and additional tumor types.

The ICD effect of cuproptosis involves several mechanisms (32). Severe mitochondrial dysfunction and proteotoxic stress during cuproptosis can induce endoplasmic reticulum (ER) stress and the unfolded protein response (UPR); ER stress is a known upstream event for CRT exposure on the cell surface (35). Mitochondrial damage leads to mitochondrial DNA (mtDNA) release, which directly activates the cGAS-STING pathway -- cyclic GMP-AMP synthase (cGAS), first identified as a cytosolic DNA sensor by Sun et al. (36), catalyzes synthesis of the second messenger cGAMP upon activation. cGAMP binds and activates STING, driving type I IFN production via the TBK1-IRF3 axis (37, 38). This pathway links ICD to adaptive immune activation. These ICD mechanisms share cGAS-STING pathway components with other ICD-inducing modalities (e.g., anthracycline chemotherapy, radiotherapy), suggesting that cuproptosis-activated ICD may follow partially conserved immune activation pathways (31). CRT exposure to the cell surface may also be mediated through the PERK-eIF2alpha-ER stress pathway, while ATP release depends partly on autophagy and pannexin-1 channels. Whether protein aggregates formed by cuproptosis-induced lipoylated protein aggregation (e.g., DLAT) can directly serve as immunostimulatory danger signals remains an untested hypothesis.

Dendritic Cell Recognition of Cuproptotic Tumor Cells Tumor immune infiltration after cuproptosis induction was analyzed using mass cytometry (CyTOF); cuproptosis induction was found to increase intratumoral MHC II+ DC and mature DC subset proportions (10). Supernatant from cuproptotic tumor cells upregulated DC maturation markers (CD40, CD80, CD86) and PD-L1 expression, and enhanced DC phagocytic capacity. In tumor-draining lymph nodes (dLN), cuproptosis induction increased conventional dendritic cell type 1 (cDC1) expansion as well as the frequency of H-2Kb-SIINFEKL-presenting DCs and tetramer+ CD8+ T cells (10, 39). DC depletion (CD11c-DTR mice + diphtheria toxin) attenuated the anti-tumor efficacy of cuproptosis inducers and eliminated tetramer+ CD8+ T cell expansion in dLN, demonstrating that DCs play an essential role in cuproptosis-driven anti-tumor T cell responses (10). The DC maturation pattern induced by cuproptosis is comparable to that induced by classical ICD inducers -- including anthracyclines (e.g., doxorubicin, mitoxantrone), oxaliplatin, and radiotherapy -- all involving type I IFN-driven cDC1 cross-presentation enhancement (28, 29, 31), though these comparisons are based primarily on indirect cross-study inference.

Evidence for cuproptosis as ICD continues to accumulate. Beyond Lei et al.’s validation in the MC38 colon cancer model (10), Disulfiram/copper has been shown to induce CRT exposure, HMGB1 release, and ATP secretion in hepatocellular carcinoma (33)—canonical ICD hallmarks—in hepatocellular carcinoma models and showed that this ICD effect enhances CD47 blockade efficacy (33), providing the first independent evidence for cuproptosis-induced ICD outside the MC38 model. Copper-induced cuproptosis was shown to elicits ICD in osteosarcoma models, where it synergizes with both radiotherapy and PD-1 blockade to suppress tumor growth (34). These data from different tumor types and different cuproptosis induction modalities provide preliminary evidence that cuproptosis-induced ICD may exhibit some cross-tumor generality. Whether cuproptosis acts as ICD in other tumor types (e.g., lung cancer, breast cancer, melanoma), and the degree to which its ICD effect depends on specific cuproptosis inducers (elesclomol-Cu2+ vs. disulfiram-Cu2+) and tumor cell metabolic backgrounds, requires further validation by multiple independent laboratories. Comparing cuproptosis with other classical ICD modalities (e.g., anthracyclines, radiotherapy) in terms of ICD quality -- including DAMP release profiling, immune memory persistence, and induced T cell receptor (TCR) repertoire diversity -- remains unexplored. Key parameters including DC activation magnitude in immunodeficient models and vaccination model protection durability require independent replication using different tumor models (10, 28). [Transition to Section 3.2: The tumor-cell-intrinsic ICD events described above are sensed by dendritic cells, which bridge cuproptosis to adaptive immunity. The following section examines how DC activation translates cuproptosis into CD8+ T cell-mediated anti-tumor responses].

### 3.2 Dendritic cell CD8+ T cell activation and TME remodeling

The TME remodeling data described in this section comes primarily from a single systematic study employing CyTOF, flow cytometry, and scRNA-seq (10). We present the findings as currently reported while noting that independent replication across different tumor models and research groups is needed to establish the generality of these observations.

The TME remodeling landscape after cuproptosis induction has been characterized using CyTOF, flow cytometry, and single-cell RNA sequencing (scRNA-seq) in the MC38 model (10, 40). Intratumoral CD8+ T cells exhibited enhanced activation (CD69+), increased proliferation (Ki67+), and augmented cytotoxicity (granzyme B+), with expansion of progenitor exhausted T cells (TPEX) without an increase in terminally exhausted T cells (TEX_term). Within CD8+ T cell subsets, certain inhibitory receptor-positive subpopulations (e.g., PD-1+ or TIM-3+) also increased, but this pattern is more consistent with activation-associated adaptive inhibitory feedback rather than terminal exhaustion (10, 41). The basic framework of copper-mediated immune cell regulation in the TME has been reviewed (11); the TME remodeling pattern after cuproptosis inducer treatment shows partial consistency with predictions from this framework.

Beyond DCs and CD8+ T cells, cuproptosis also remodeled other TME components. Activated CD4+ T cells increased, while NK cells showed no significant increase, suggesting a T cell-centric response. Tumor-associated macrophages (TAMs) exhibited an elevated M1/M2 ratio after cuproptosis induction, and cuproptotic cell-conditioned medium alone sufficed to recapitulate this polarization shift *in vitro* (42). Colony-stimulating factor 1 receptor (CSF1R) blockade enhanced the intratumoral M1/M2 TAM ratio, increased CD8+ T cell IFN-γ expression, and improved cuproptosis-mediated tumor control (10). TME remodeling involves coordinated regulation of multiple immune cell types and stromal components (40, 43).

### 3.3 Positive feedback: CD8+ T cells enhance tumor cell cuproptosis via IFN-γ-STAT1-IRF1-FDX1

The bidirectional feedback loop described in this section, in which CD8+ T cell-derived IFN-γ (44) upregulates FDX1 via STAT1-IRF1 to enhance tumor cell cuproptosis sensitivity, derives from a single study (10) and has not yet been independently replicated. We describe the mechanistic evidence here, but stress that independent validation in additional tumor models and by separate research groups is essential before this model can be considered established.

Lei et al.’s study revealed a bidirectional regulatory relationship between cuproptosis and anti-tumor immunity -- cuproptosis does not unidirectionally activate the immune system; rather, the activated immune system reciprocally enhances tumor cell sensitivity to cuproptosis, forming a positive feedback loop (10). Such bidirectional regulatory paradigms are not unique in the cell death-immunity intersection: CD8+ T cell-derived IFN-γ has been shown to promote tumor cell ferroptosis by downregulating SLC7A11 and SLC3A2 (45), providing a parallel paradigm in the ferroptosis field. However, cuproptosis and ferroptosis exhibit mechanistic differences in their respective IFN-γ feedback loops: the ferroptosis loop operates through SLC7A11 downregulation and cystine deprivation, whereas the cuproptosis loop proceeds through FDX1 transcriptional upregulation and copper sensitivity enhancement, reflecting the distinct molecular wiring of the two death modes.

At the mechanistic level, IFN-γ secreted by activated CD8+ T cells binds to IFNGR1/IFNGR2 receptors on tumor cells, activating JAK1/JAK2 kinases, which phosphorylate STAT1. Phosphorylated STAT1 dimerizes, translocates to the nucleus, and directly binds to the IRF1 promoter to drive its transcription (10, 46). Integrated ChIP-seq and RNA-seq analyses demonstrated that IRF1 directly occupies the FDX1 gene promoter and activates FDX1 transcription (10). This STAT1-IRF1-FDX1 transcriptional cascade explains why IFN-γ enhances tumor cell sensitivity to cuproptosis: FDX1 upregulation increases the capacity to reduce Cu2+ to Cu+, and FDX1 itself is a direct cuproptosis effector target (4, 10). IFN-γ pretreatment of tumor cells synergistically enhanced elesclomol-Cu2+-induced cell death, and this synergy was abolished by FDX1 knockdown or copper chelator BCS treatment (10).

This positive feedback loop holds two immunological implications. First, it constitutes a self-amplifying circuit: cuproptosis -> ICD -> DC maturation -> CD8+ T cell activation -> IFN-γ secretion -> STAT1-IRF1 -> FDX1 upregulation -> enhanced cuproptosis sensitivity. Second, it provides a molecular explanation for the synergy between cuproptosis induction and immune checkpoint inhibitor (ICI) therapy: tumor-infiltrating CD8+ T cells producing IFN-γ sensitize tumor cells to cuproptosis while simultaneously inducing PD-L1 expression, creating conditions where combined anti-PD-L1 and cuproptosis induction may produce supra-additive anti-tumor effects (14, 46–50).

The question of whether the IFN-γ-STAT1-IRF1-FDX1 axis exhibits cancer cell selectivity warrants attention. IFN-γ was found to induce only modest FDX1 expression in non-cancer cells (10), including mouse embryonic fibroblasts (MEFs), human mammary epithelial cells (MCF10A), and primary human CD8+ T cells (10). This differential response provides a mechanistic basis for a therapeutic window between tumor and normal cells. However, this selectivity currently rests on data from a single study and a single experimental system; its stability under different cuproptosis induction conditions and tumor models requires independent validation. In independent experiments, PD-L1 blockade enhanced intratumoral CD8+ T cell IFN-γ expression, upregulating IRF1 and FDX1, thereby overcoming intrinsic and acquired resistance to PD-L1 blockade and directly extending and consolidating Lei et al.’s core findings (10).

In independent work, A ROS-responsive nanoparticle system for co-delivery of elesclomol and Cu combined with anti-PD-L1 demonstrated cuproptosis-induced DC maturation, enhanced CD8+ T cell tumor infiltration, and synergistic tumor suppression in B16-F10 melanoma and 4T1 breast cancer models (47). A copper oxide nanoplatform for elesclomol delivery was shown to trigger cuproptosis and enhance anti-tumor immunotherapy, with confirmed increases in DC maturation (CD80/CD86 upregulation) and CD8+ T cell tumor infiltration (48). These independent data from different research groups provide additional multi-model evidence for synergistic effects between cuproptosis induction and ICI. These studies confirm the ICD effect and immunotherapeutic synergy of cuproptosis, but none independently validates the STAT1-IRF1-FDX1 transcriptional cascade that constitutes the mechanistic core of the positive feedback loop; independent replication of this molecular mechanism remains an outstanding need.

## 4 Potential immunosuppressive risks of cuproptosis

Although cuproptosis has yielded preliminary experimental evidence for promoting anti-tumor immunity, the pleiotropic effects of copper overload on the immune system mean that this effect may not be unidirectional. This section discusses the potential immunosuppressive risks accompanying cuproptosis, with strict distinction between two categories: (I) direct toxicity of copper ions toward immune cells -- this falls within the pharmacological effects of copper and does not equal immunological consequences arising from the cuproptosis induction process; (II) immunosuppressive signals potentially accompanying the cuproptosis induction process -- this is a cuproptosis-specific issue as a cell death mode. In the existing literature, category (I) has relatively rich indirect evidence (primarily from copper nutritional immunology and copper chelation studies), while category (II) is currently based mainly on theoretical extrapolation and borrowing from adjacent fields such as ferroptosis. This section addresses each category separately, and where direct experimental data are lacking, the evidence level is explicitly noted.

### 4.1 Direct toxicity of copper ions toward immune cells: distinguishing pharmacological effects from cuproptosis induction

Copper is an essential trace element for normal immune cell function, but the concentration boundary between nutritional requirement and toxicity is narrow. Following T cell receptor (TCR) signaling activation, CTR1-mediated copper influx contributes to IL-2 production and T cell proliferation at physiological copper levels (49, 50). When copper concentrations exceed buffering capacity, however, copper-catalyzed Fenton-like reactions generate hydroxyl radicals that damage lymphocyte membranes and intracellular proteins (49). Copper can also displace other metals from metalloproteins critical for lymphocyte function, including zinc finger transcription factors involved in T cell development and activation. This dual role of copper in T cell biology—required for activation at low concentrations but toxic at elevated concentrations—underscores the challenge of achieving selective tumor cell cuproptosis without impairing anti-tumor immunity.

Beyond direct toxicity effects, Copper maintains regulatory T cell (Treg) immunosuppressive function by supporting oxidative phosphorylation metabolism (51). This study showed that Treg cells may have greater metabolic dependence on copper than effector T cells; copper deficiency can impair Treg suppressive function while copper excess may potentiate Treg-mediated immunosuppression. This finding provides a new mechanistic perspective for understanding copper’s immunological bidirectional effect in the TME: excess copper delivered by cuproptosis inducers may, while damaging effector T cells, produce immunosuppressive effects by maintaining or enhancing Treg function, thereby partially offsetting the immune activation from ICD.

In the cuproptosis context, the core question regarding this bidirectional effect is the therapeutic window: whether a copper ion concentration range exists that reliably triggers tumor cell cuproptosis while sparing immune cell function. Recent data complicate this picture: Copper has been shown to support Treg energetic metabolism and is required for peripheral immune tolerance maintenance (51). Copper ionophore therapy could prove immunosuppressive if it impairs effector T cells while simultaneously supporting Treg function. The therapeutic window for copper-based immunotherapy has not been experimentally defined for any tumor model. Dose-response curves for different immune cell subsets (CD8+ T cells, CD4+ T cells, NK cells, Tregs, dendritic cells) exposed to elesclomol-Cu2+ or disulfiram-Cu2+ are needed to determine whether a viable therapeutic window exists, and whether this window varies across tumor types and immune contexts.

### 4.2 Spatial heterogeneity of copper distribution in the tumor microenvironment

Copper distribution within tumor tissue is not homogeneous. Metal element spatial imaging studies using laser ablation-inductively coupled plasma-mass spectrometry (LA-ICP-MS) show that copper concentrations in tumor central regions are often lower than those at the invasive front and tumor-stroma interface (16). This spatial heterogeneity means that after systemic administration, copper delivered by copper ionophores may reach different local concentrations in different tumor regions -- high-copper regions at the invasive front may produce more effective cuproptosis and ICD, while low-copper regions in the tumor center may have insufficient cuproptosis induction. Simultaneously, immune infiltration density in different tumor regions itself exhibits significant spatial heterogeneity (39, 40). The superposition of these two heterogeneities -- the interaction between copper distribution gradients and immune cell distribution gradients -- makes predicting the net immunological effect of cuproptosis induction in specific tumor regions difficult. LA-ICP-MS data currently derive primarily from baseline tumor samples without cuproptosis induction treatment; systematic collection of dynamic changes in copper spatial distribution after treatment remains needed.

### 4.3 Immunosuppressive signals potentially accompanying cuproptosis: extrapolation from ICD and ferroptosis fields

Although cuproptosis-induced tumor cell death has been preliminarily confirmed as immunogenic (10), whether the factors released by dying or stressed cells include immunosuppressive components remains an open question. ICD is known to accompany the release of immunosuppressive cytokines -- such as interleukin-10 (IL-10) and transforming growth factor-beta (TGF-beta) -- which, under certain conditions, can shift the overall balance of immune responses toward tolerance (28, 43). Whether cuproptotic cell-conditioned medium is enriched in these immunosuppressive factors, and whether their concentrations are sufficient to alter T cell function *in vivo*, currently lacks direct experimental data in cuproptosis models. Indirect evidence from related cell death fields provides preliminary support: IL-10 release has been documented in ferroptotic tumor cells (23, 52), and TGF-beta activation has been observed upon copper-overload-induced oxidative stress in multiple cell types, including hepatic stellate cells and renal tubular epithelial cells. These findings from ferroptosis and copper toxicity models suggest that cuproptotic cells may also release immunosuppressive cytokines, though direct measurement in cuproptosis-conditioned medium is needed for confirmation. The complete DAMP spectrum released during cuproptosis -- including which protein aggregates, metabolites, and lipid mediators may exert immunomodulatory activity -- has also not been identified. This gap is a core obstacle to assessing the net direction of cuproptosis immunological effects. Different tumor cell types may release highly heterogeneous compositions of immunomodulatory factors after undergoing cuproptosis, further increasing uncertainty.

Intratumoral TAM polarization direction is a key determinant of the immunological outcome of cuproptosis. Although cuproptosis has been shown to drive M1-type TAM polarization and improve anti-tumor immunity (10), TAM polarization after cuproptosis induction may be highly heterogeneous across different tumor models and patients. In the ferroptosis field, studies have reported that oxidized lipid mediators released by dying cells can induce TAMs toward M2 polarization (23, 42). Whether the immunostimulatory effects of cuproptosis-induced lipoylated protein aggregates and mtDNA are attenuated or subverted by the immunosuppressive network of the TME *in vivo* remains an open question. Whether cuproptosis or copper overload affects MDSC recruitment and function -- for example, through copper-induced chemokine upregulation (e.g., CCL2) or through direct effects of copper on MDSC metabolism -- currently has very limited evidence (53, 54), based primarily on general principles of copper nutritional immunology rather than cuproptosis-specific experimental data.

In terms of immune activation, Inhibition of NPLOC4, one of the targets of the disulfiram-Cu complex, drives TAM reprogramming from M2 to M1, synergizing with anti-PD-1 therapy (55). Although this study did not directly use cuproptosis inducers, it suggests that intervention at copper metabolism-related targets may enhance anti-tumor immunity through remodeling TAM polarization, providing a new conceptual framework for cuproptosis-mediated indirect modulation of TME immune activation. This study currently provides preliminary evidence for TAM reprogramming direction, and its direct relevance to cuproptosis induction requires validation.

## 5 Core controversies and open questions in cuproptosis-immunity research

### 5.1 The immunogenicity of cuproptosis: universal principle or condition-dependent?

Lei et al.’s study in Cell confirmed that cuproptosis meets all gold-standard ICD criteria in the MC38 colon cancer model (10). As reviewed in Section 3.1, accumulating independent ICD evidence has provided preliminary cross-model support in multiple tumor types -- though complete ICD gold-standard validation remains primarily limited to the MC38 colon cancer model. The capacity of cuproptosis to induce ICD depends at minimum on: (1) the type of copper ionophore (elesclomol vs. disulfiram vs. other copper carriers), as different carriers may differ in copper delivery efficiency and subcellular targeting; (2) the metabolic background of tumor cells -- FDX1 expression level, lipoylation activity, and mitochondrial respiratory chain integrity, with highly OXPHOS-dependent tumors (e.g., certain melanoma and pancreatic cancer subtypes) potentially more sensitive to cuproptosis-induced ICD than highly glycolytic tumors; (3) host immune status -- the anti-tumor efficacy of cuproptosis observed in immunocompetent mice is attenuated in immunodeficient settings (10), suggesting that cuproptosis-induced ICD may only produce clinically meaningful anti-tumor effects in immune-competent hosts; (4) copper formulation -- different copper formulations (CuCl2, copper gluconate, copper nanoparticles) differ in bioavailability and tissue distribution, which may affect ICD induction quality. Systematic deconstruction of these condition dependencies is essential for moving the field from phenomenological reporting to mechanistic understanding.

### 5.2 The immune activation window of copper ions

The relationship between copper ion concentration and immunological effect is not linear. Low copper concentrations support immune function as an essential cofactor; moderate copper concentrations may activate cGAS-STING and type I IFN pathways through mild mitochondrial stress induction; high copper concentrations directly kill immune cells. However, the concentration boundaries of these three zones, their exposure-time dependence, and tumor-type specificity all lack systematic quantitative data. Half-maximal inhibitory concentrations (IC50) and selectivity indices (SI = tumor cell IC50/immune cell IC50) of copper ions for different immune cell subsets including CD8+ T cells, CD4+ T cells, NK cells, DCs, and TAMs require determination in standardized experimental systems. The impact of dynamic changes in *in vivo* copper protein binding ratios (ceruloplasmin-, albumin-, transcuprein-bound copper vs. free copper ratios) on actual immune cell exposure before and after cuproptosis induction treatment currently lacks pharmacokinetic-pharmacodynamic (PK-PD) modeling studies.

### 5.3 Comparative immunological effects of cuproptosis vs. ferroptosis

This section’s comparison of ferroptosis and cuproptosis immunological effects rests on a fundamental evidence asymmetry: ferroptosis-ICD research has accumulated extensive multi-laboratory independent data, while cuproptosis-ICD -- despite preliminary supplementation from hepatocellular carcinoma (33) and osteosarcoma (34) models -- has a smaller sample size. The following comparison should therefore be regarded as a hypothesis framework requiring experimental testing, rather than established conclusions.

Ferroptosis has been confirmed to possess ICD-inducing capacity, and ferroptosis-ICI combination therapy has shown synergistic effects in multiple independent preclinical models (23, 27, 56, 57). CD8+ T cell-derived IFN-γ has been shown to promote tumor cell ferroptosis through downregulation of SLC7A11 (41) and SLC3A2 (45); this discovery and Lei et al.’s IFN-γ-STAT1-IRF1-FDX1 axis form parallel paradigms across death modes, suggesting that bidirectional regulation between metal-dependent cell death and adaptive immunity may be a more general biological principle. However, cuproptosis and ferroptosis may differ importantly in the following immunological dimensions: (1) DAMP release profile: ferroptosis primarily releases HMGB1, ATP, and oxidized lipids (e.g., oxidized phosphatidylethanolamines), whereas cuproptosis, in addition to sharing some DAMPs, may additionally release mtDNA and lipoylated protein aggregates (10, 23); (2) DC activation mode: ferroptosis-induced DC activation partially depends on the lipid peroxide-TLR4 pathway, whereas cuproptosis depends more on the mtDNA-cGAS-STING-type I IFN axis (10, 23); (3) immune memory: ferroptosis-induced immune memory has been validated in multiple independent prophylactic vaccination models (24), while cuproptosis immune memory data are currently limited to (10); (4) mechanism of ICI synergy: ferroptosis-ICI synergy operates partly through PD-L1 downregulation and enhanced T cell infiltration, while cuproptosis synergy involves PD-L1 upregulation -- a distinct mechanistic logic (**Table 1**).

### 5.4 The biomarker challenge in cuproptosis

A core bottleneck in translating cuproptosis-targeted therapy to clinical practice lies in biomarkers. An ideal cuproptosis predictive biomarker should answer two questions: first, whether a given patient’s tumor is sensitive to cuproptosis induction (predicting therapeutic efficacy); second, whether, after cuproptosis induction, the tumor microenvironment has undergone changes favoring immune responses (predicting immune synergy).

The FDX1-lipoylated protein axis is currently the biomarker source with the strongest evidential support. Tumor cells with high FDX1 expression are more sensitive to cuproptosis; thus, FDX1 immunohistochemistry (IHC) scoring may serve as an efficacy predictive marker (4, 10, 17). However, the relationship between FDX1 mRNA expression and protein-level lipoylation activity is not simply linear -- lipoylation completion also depends on LIAS activity and Fe-S cluster biosynthesis, increasing the uncertainty of prediction based solely on FDX1 transcript levels. The integrity of the STAT1-IRF1-FDX1 axis further extends the biomarker dimension: IRF1 binding site mutation/deletion status may be a key supplementary dimension for predicting IFN-γ-enhanced cuproptosis sensitivity (10), though this genetic marker requires validation in larger patient cohorts. Cuproptosis-related prognostic signatures based on multi-gene expression profiles have been explored in bioinformatics analyses across multiple tumor types (e.g., clear cell renal cell carcinoma (58)), but their clinical utility as predictive markers for cuproptosis-targeted therapy has not been evaluated in prospective studies. The negative results of the SYMMETRY trial serve as a reminder: without validated biomarker stratification, even mechanistically plausible targeted therapies can yield negative clinical trial results (59).

### 5.5 Methodological limitations of current research and directions for improvement

Current cuproptosis immunological research relies primarily on the following experimental systems, each with its advantages and limitations; none captures the full complexity of human tumor-immune interactions. (1) Immunodeficient mouse xenograft models: These models lack adaptive immune components and cannot evaluate cuproptosis-induced ICD effects, providing only direct tumor cell killing data. (2) Immunocompetent syngeneic transplant models (e.g., MC38, B16-F10, CT26): such models have been employed to reveal cuproptosis-immunity bidirectional regulation (10), but syngeneic transplant growth kinetics and immune microenvironment characteristics differ from spontaneous tumors. (3) Patient-derived xenograft (PDX) models: PDX models preserve patient tumor heterogeneity and stromal architecture but require establishment in immunodeficient mice and cannot directly assess ICD. Humanized mouse models are emerging as a tool to bridge this gap but currently face limitations including variable engraftment efficiency and graft-versus-host disease. (4) Genetically engineered mouse models (GEMMs): Spontaneous tumors developing in immunocompetent hosts through natural immune editing represent the closest-to-clinical-reality model for assessing cuproptosis-immunity relationships but have not been used in the cuproptosis field. (5) Bioinformatics analysis of public databases: While cuproptosis-related gene expression profiling based on TCGA and other databases can provide large-scale correlational clues, such analyses have inherent limitations -- correlation does not imply causation, and bulk RNA-seq cannot resolve cell-type-specific expression -- meaning that conclusions must be validated in functional experiments. The cuproptosis-related gene signatures commonly used in bioinformatics studies lack unified standards in gene membership and algorithms (58), increasing the difficulty of cross-study comparability.

## 6 Translational prospects and clinical strategies

### 6.1 Combination regimen design for copper ionophores and immunotherapy

Among existing copper ionophores (56, 57, 60, 61), elesclomol and disulfiram have the clearest clinical translational prospects.

Elesclomol is a selective copper ionophore whose mechanism of specifically transporting copper to mitochondria to induce oxidative stress was elucidated in early studies (30). The drug was evaluated in a Phase III clinical trial (SYMMETRY study) in patients with advanced melanoma. Although this trial failed to meet its primary endpoint of progression-free survival (PFS), *post hoc* analysis indicated that patients with normal baseline serum lactate dehydrogenase (LDH) may derive benefit from elesclomol plus paclitaxel (59). The SYMMETRY trial also provided important safety data for elesclomol: grade 3–4 adverse events were primarily neutropenia, peripheral sensory neuropathy, and fatigue, with neurotoxicity being elesclomol’s characteristic dose-limiting toxicity. When elesclomol is combined with ICI, careful assessment is needed regarding whether neurotoxicity may overlap or synergistically amplify with ICI-related immune-related adverse events (e.g., immune-related neuropathy). Pharmacokinetic data from the SYMMETRY trial showed elesclomol’s short half-life (approximately 0.9 hours), which favors temporal optimization in combination dosing regimens to limit the copper exposure window within a controllable range. Given that elevated LDH is a marker of tumor hypoxia and metabolic reprogramming, and that FDX1-lipoylation axis activity is closely related to mitochondrial metabolic status, FDX1 expression-based patient stratification may help rediscover elesclomol’s clinical value -- particularly in the new context of ICI combination. Translation from mouse models to clinical efficacy still requires rigorous clinical trial validation, and the negative results of the SYMMETRY trial demonstrate that positive preclinical model results do not always predict clinical efficacy. Future clinical trial designs should include: (1) IHC stratification for FDX1 and lipoylated proteins; (2) pharmacodynamic endpoints (such as dynamic changes in serum mtDNA, IFN-beta, and ceruloplasmin levels); (3) prespecified immune monitoring endpoints (including peripheral blood T cell subsets, DC maturation markers, and tumor biopsy immune infiltration analysis). The feasibility of elesclomol-Cu in nano-delivery systems combined with ICI has been further confirmed (47, 48).

Disulfiram is an FDA-approved alcohol-aversion drug that forms a CuET complex with Cu2+ *in vivo*, exerting anti-cancer activity by targeting the p97/VCP-NPL4 pathway (62). Whether CuET’s anti-cancer mechanism partially proceeds through the cuproptosis pathway remains an open question, but disulfiram’s established safety data and low cost support its candidacy as a cuproptosis-immune combination therapy agent. Studies are exploring the efficacy of disulfiram/Cu combined with anti-PD-1 in preclinical models.

In independent experimental systems, Disulfiram combined with anti-PD-L1 abrogates NSCLC drug resistance through ATP7B downregulation and HIF-1α signaling pathway modulation (63). A liposomal delivery strategy for disulfiram metabolites has confirmed that copper-mediated tumor immune activation can be optimized through nanocarriers (64). Independent validation in hepatocellular carcinoma models demonstrated that disulfiram/copper induces ICD and enhances CD47 blockade efficacy (33), providing preliminary evidence from independent laboratories for disulfiram/copper as a cuproptosis-immune combination therapy candidate. Disulfiram/copper activates the cGAS-STING innate immune pathway through ROS-induced DNA damage, contributing to anti-tumor immunity independently of cuproptosis-induced ICD effects, providing an additional mechanistic rationale for drug combination design (65). demonstrated that disulfiram/copper activates the cGAS-STING innate immune pathway through ROS-induced DNA damage, and that this activation contributes to anti-tumor immunity independently of classical cuproptosis-mediated cell death (65). This finding suggests copper ionophores may engage multiple immunostimulatory pathways—both cuproptosis-dependent ICD and direct cGAS-STING activation—potentially broadening their therapeutic applicability.

Dosing sequence of combination regimens is another critical variable requiring optimization. The theoretical advantage of sequential regimens (cuproptosis induction followed by immunotherapy) is that immunogenic inflammation is first created, then immunosuppression is relieved with ICI; concurrent administration may compromise ICI efficacy through copper-mediated inhibition of activated T cells. However, concurrent administration of copper ionophores with ICI has shown synergistic effects (10), suggesting that with appropriate dose and formulation design, concurrent combination regimens may achieve maximal synergy. Differential combinations of copper ionophore dose, copper formulation (CuCl2 vs. copper gluconate), and immune checkpoint inhibitor type still require systematic comparison in more *in vivo* studies.

### 6.2 Nano-delivery systems for tumor-targeted copper enrichment

Nano-delivery (66, 67) offers one strategy to address systemic copper exposure and immunotoxicity, though clinical translation of nano-formulations faces manufacturing and regulatory hurdles. Copper-based nanoparticles (e.g., copper-metal-organic frameworks Cu-MOF, copper sulfide nanoparticles CuS NP, cuprous oxide nanoparticles) can achieve selective copper enrichment in tumor tissue while reducing effects on circulating immune cells (68). Some copper-based nanoplatforms also possess photothermal/photodynamic therapeutic functions, enabling localized hyperthermia and ROS burst under near-infrared light irradiation, further enhancing cuproptosis and ICD effects (68). Surface functionalization of nanoparticles with targeting ligands (e.g., folate, transferrin, RGD-containing peptides) can improve tumor specificity and cellular internalization efficiency. Nano-delivery strategies introduce additional complexity. The intrinsic immunogenicity of copper-based nanocarriers -- whether the nanomaterials themselves activate complement systems, induce anti-PEG antibody production, or trigger excessive clearance by the mononuclear phagocyte system -- is an important safety dimension requiring evaluation (66). Nanoparticle accumulation in the liver and spleen may produce long-term effects on tissue-resident immune cells (e.g., Kupffer cells and splenic marginal zone macrophages); the development of degradable copper-based nanoplatforms (e.g., pH-responsive Cu-MOF) is designed to address this challenge (67, 68). Despite these challenges, nano-delivery strategies can, at the pharmacokinetic level, broaden the therapeutic window of cuproptosis induction in addressing the immunological bidirectional effect discussed in Section 4.

Recent reviews have covered cuproptosis-immunotherapy synergistic nanomedicine design strategies (69), cuproptosis-based metabolic reprogramming and nanomedicine approaches (70), and the roles of copper homeostasis and cuproptosis in tumor immunity (71). These reviews offer technical roadmaps for the potential application of copper-based nano-delivery systems in cuproptosis-immune combination therapy.

### 6.3 Cuproptosis biomarker-based patient stratification

The success of cuproptosis-immune combination therapy critically depends on rational patient stratification strategies. A proposed stratification framework includes three dimensions:

1. Tumor cuproptosis sensitivity assessment: IHC or mRNA expression levels of FDX1, LIAS, and DLAT, combined with serum ceruloplasmin and tumor tissue copper content measurements. Lei et al.’s study further suggests that assessment of STAT1-IRF1-FDX1 axis integrity -- including IRF1 binding site mutation/deletion status -- may be a key supplementary dimension for predicting IFN-γ-enhanced cuproptosis sensitivity (10).
2. Baseline immune microenvironment status: Tumor-infiltrating lymphocyte (TIL) density, PD-L1 expression, tumor mutational burden (TMB), and microsatellite instability (MSI) status.
3. Copper metabolism gene mutations/copy number variations: SLC31A1 amplification, ATP7A/ATP7B deletion, etc., may indicate reduced tumor tolerance to copper accumulation, making them more suitable for cuproptosis induction strategies.

Based on existing evidence, tumors with high FDX1 expression, intact STAT1-IRF1 signaling, low-to-moderate TILs, and low/negative PD-L1 may represent the optimal population for cuproptosis/ICD-ICI sequential regimens, as cuproptosis-induced ICD can convert such “cold tumors” into ICI-responsive tumors (10). However, the validity of this stratification strategy requires verification in prospective clinical trials.

### 6.4 Distinguishing clinical scenarios for copper chelation vs. copper overload

Cuproptosis induction and copper chelation represent two extreme directions in copper metabolism-targeted therapy. A preliminary decision framework can be summarized as: (1) Suitable for cuproptosis induction strategy (copper overload): tumors with high FDX1 expression, high lipoylation activity, high SLC31A1 expression or copper efflux defects, and high mitochondrial metabolic activity (4, 10, 16, 72, 73); (2) Suitable for copper chelation strategy: tumors with copper accumulation-dependent angiogenesis (e.g., certain breast and hepatocellular carcinomas) (3, 74), high PD-L1 expression positively correlated with copper levels (13, 74); clinical studies show that the copper chelator tetrathiomolybdate can reduce circulating endothelial progenitor cell numbers in patients at high risk for breast cancer recurrence and showed preliminary evidence of affecting the tumor microenvironment in a Phase II clinical trial (74); (3) Not suitable for any copper metabolism intervention: tumors with severe copper homeostasis defects caused by loss-of-function mutations in copper metabolism-related genes. This decision framework is currently a preliminary extrapolation based on existing preclinical and early clinical evidence and requires testing in prospective clinical studies (75).

### 6.5 New target discovery and drug development directions

Understanding cuproptosis-immune intersection mechanisms has driven the following drug development directions: (1) STING agonists combined with copper ionophores: exploiting mtDNA release during cuproptosis to provide endogenous activation signals for the STING pathway, combined with exogenous STING agonists (e.g., ADU-S100, MK-1454), may produce synergistic immune activation effects -- this strategy has preliminary preclinical data support but has not been validated in a cuproptosis-specific context. (2) Cuproptosis-specific immune checkpoints (speculative direction): whether new immune checkpoint molecules are exposed on the tumor cell surface during cuproptosis (analogous to CD47 function on apoptotic cells) is currently theoretical speculation without any experimental data support. Identification of such molecules through proteomic screening would open an entirely new therapeutic target space in the cuproptosis-immunity field. (3) IRF1/FDX1-activating small molecules: given that the STAT1-IRF1-FDX1 axis is the key effector of IFN-γ-enhanced cuproptosis sensitivity (10), developing IRF1 or FDX1 expression upregulators could be a strategy to enhance tumor cuproptosis sensitivity. This direction depends on high-throughput screening of IRF1/FDX1 small-molecule agonists, for which no publicly reported medicinal chemistry studies currently exist. (4) Cuproptosis and chimeric antigen receptor T cell (CAR-T) combination (speculative direction): cuproptosis preconditioning may remodel the immunosuppressive landscape of the tumor microenvironment, creating more favorable conditions for CAR-T cell infiltration and effector function, but this hypothesis currently derives entirely from general extrapolation of ICD-CAR-T combination strategies, without direct experimental validation in the cuproptosis field. (5), sequential combination strategy of cuproptosis and copper chelation: the immunogenic inflammation produced after cuproptosis induction can remodel the TME; sequential application of copper chelators may weaken immunosuppression by reducing Treg copper-dependent metabolic support while preserving the ICD effect induced by cuproptosis, producing synergistic immunological effects. This strategy is currently at the conceptual stage (**Table 3**), but its theoretical basis has received preliminary support in independent literature (10, 51, 74).

**Table 3:** Current clinical translational advances in cuproptosis-based cancer immunotherapy.

Strategy	Agent/platform	Mechanism	Stage & evidence	Key reference
Copper ionophore + ICI	Elesclomol-Cu + anti-PD-L1	Cuproptosis ICD + checkpoint blockade	Preclinical (MC38, B16)	Lei et al., 2026 (10)
Nanoplatform + ICI	Cu2O@MSN-ELC + anti-PD-L1	Tumor-targeted copper release + ICD	Preclinical (4T1 breast)	Lu et al., 2024 (48)
ROS-responsive NP + ICI	ROS-NP(ELC/Cu) + anti-PD-L1	TME-specific copper delivery	Preclinical (CT26 colon)	Guo et al., 2023 (47)
DSF-Cu + PD-1 blockade	DSF-Cu + anti-PD-1	NPL4 inhibition + cGAS-STING	Preclinical (MC38, LLC)	Yuan et al., 2025 (65)
DSF-Cu + CD47 blockade	DSF-Cu + anti-CD47	ICD + macrophage checkpoint	Preclinical (HCC)	Gao et al., 2022 (33)
Liposomal DSF metabolite	Liposomal DTC-Cu	Intratumoral copper + T cell immunity	Preclinical (B16F10)	Heroux et al., 2025 (64)
Cuproptosis + radiotherapy	Copper + RT + PD-1 blockade	Cuproptosis ICD + RT synergy	Preclinical (osteosarcoma)	Yao et al., 2025 (34)
Copper chelation	Tetrathiomolybdate	Copper depletion in high-risk BC	Phase II NCT00195091	Chan et al., 2017 (74)
Elesclomol + paclitaxel	Elesclomol + paclitaxel	Cuproptosis + chemotherapy	Phase III SYMMETRY NCT00522834 (negative)	O’Day et al., 2013 (59)
DSF-Cu + anti-PD-L1	DSF-Cu + anti-PD-L1	ATP7B-HIF-1 modulation	Preclinical (NSCLC)	Li et al., 2024 (63)

## 7 Conclusions and perspectives

Cuproptosis was described as a distinct form of programmed cell death in 2022. Since then, research at its intersection with anti-tumor immunity has moved from describing phenotypes toward understanding mechanisms. This review examined the evidence for bidirectional regulation between cuproptosis and anti-tumor immunity and assessed the strength and limitations of the current experimental data.

Cuproptosis activates DC-dependent cross-presentation, expands antigen-specific CD8+ T cells, and remodels the tumor microenvironment through ICD. Activated CD8+ T cells, in turn, secrete IFN-γ, which drives STAT1-IRF1-mediated FDX1 upregulation, sensitizing tumor cells to further cuproptosis. This positive feedback loop parallels the CD8+ T cell-IFN-γ-ferroptosis circuit described by Wang et al. (45)., suggesting that metal-dependent cell death and adaptive immunity may engage in recurrent regulatory motifs across death modes. The core data for this cuproptosis-immunity loop come from a single systematic study by Lei et al.; its ICD component has received preliminary corroboration from independent laboratories using hepatocellular carcinoma and osteosarcoma models. Whether the complete IFN-γ-STAT1-IRF1-FDX1 loop operates across diverse tumor types and induction conditions remains to be independently confirmed.

Copper ion toxicity toward T cells and NK cells, spatial heterogeneity of intratumoral copper distribution, and potential release of immunosuppressive signals all suggest that the net immunological effect of cuproptosis is likely more nuanced than current ICD evidence alone indicates. Some of these concerns, such as direct copper immunotoxicity, have independent literature support, while others, including cuproptosis-associated immunosuppressive cytokine release, remain extrapolations. Distinguishing evidence-grounded risks from speculative ones, as detailed in Section 4, is needed before evaluating the clinical feasibility of cuproptosis-inducing strategies.

Several lines of investigation would help move the field forward. First, independent validation of cuproptosis immunogenicity across tumor types, using syngeneic transplant models, patient-derived xenografts, and genetically engineered mouse models in multi-center studies, is needed to establish the generalizability that clinical translation requires. Second, quantitative definition of the copper dose-immunological effect relationship, with dose-response curves and selective toxicity thresholds measured for distinct immune cell subsets across tumor types, would address the immunological bidirectionality problem discussed in Section 4. Third, development and clinical validation of cuproptosis biomarkers, particularly protein-level assessments of FDX1-STAT1-IRF1 axis integrity and lipoylation status rather than mRNA expression alone, is essential; the negative outcome of the SYMMETRY trial illustrates that even mechanistically well-supported therapies can fail without effective patient stratification. Fourth, immunologic comparison of cuproptosis with ferroptosis and other metal-dependent death modes, using unified prophylactic vaccination models, DAMP release profiling, TCR clonal diversity analysis, and single-cell TME mapping, would inform both mechanistic understanding and clinical decisions about which death mode to target. Finally, cross-scale integration, from structural biology of FDX1-Cu-lipoylated protein interactions, through single-cell multi-omics of TME remodeling after cuproptosis, to whole-animal immunopharmacology under diverse immune backgrounds, is needed to bridge the evidence gap between mechanism and clinical application.

As independent validation data accumulate, biomarker-guided trials progress, and nano-delivery technologies mature, the place of cuproptosis in tumor immunotherapy will sharpen. The field currently sits between early mechanistic discoveries and clinical readiness; closing the critical experimental gaps identified here will determine how quickly and in what form it reaches the latter.
